# Detection of decreased glomerular filtration rate in intensive care units: serum cystatin C *versus* serum creatinine

**DOI:** 10.1186/1471-2369-15-9

**Published:** 2014-01-13

**Authors:** Pierre Delanaye, Etienne Cavalier, Jérôme Morel, Manolie Mehdi, Nicolas Maillard, Guillaume Claisse, Bernard Lambermont, Bernard E Dubois, Pierre Damas, Jean-Marie Krzesinski, Alexandre Lautrette, Christophe Mariat

**Affiliations:** 1 Department of Nephrology-Dialysis, University of Liège, CHU Sart Tilman, Liège 4000, Belgium; 2 Department of Clinical Chemistry, University of Liège, CHU Sart Tilman, Belgium; 3 Department of General Intensive care, University Jean Monnet, Hôpital Nord, Saint-Etienne, France; 4 Department of Nephrological Intensive Care, University Jean Monnet, Hôpital Nord, Saint-Etienne, France; 5 Department of Medical Intensive Care, University of Liège, CHU Sart Tilman, Belgium; 6 Department of General Intensive care, University of Liège, CHU Sart Tilman, Belgium; 7 Department of Intensive Care Unit, University of Clermont-Ferrand, Clermont-Ferrand, France

**Keywords:** Kidney failure, Cystatin C, Creatinine

## Abstract

**Background:**

Detecting impaired glomerular filtration rate (GFR) is important in intensive care units (ICU) in order to diagnose acute kidney injuries and adjust the dose of renally excreted drugs. Whether serum Cystatin C (SCysC) may better reflect glomerular filtration rate than serum creatinine (SCr) in the context of intensive care medicine is uncertain.

**Methods:**

We compared the performance of SCysC and SCr as biomarkers of GFR in 47 critically ill patients (median SOFA (Sepsis-related Organ Failure Assessment) score of 5) for whom GFR was measured by a reference method (urinary clearance of iohexol).

**Results:**

Mean Iohexol clearance averaged 96 ± 54 mL/min and was under 60 mL/min in 28% of patients. Mean SCr and SCysC concentrations were 0.70 ± 0.33 mg/dL and 1.26 ± 0.61 mg/L, respectively. Area under the ROC curve for a GFR threshold of 60 mL/min was 0.799 and 0.942 for SCr and SCysC, respectively (p = 0.014).

**Conclusions:**

We conclude that ScysC significantly outperfoms SCr for the detection of an impaired GFR in critically ill patients.

**Trial registration:**

ClinicalTrials.gov: B7072006347

## Background

Detecting impaired glomerular filtration rate (GFR) is important in intensive care units (ICU) in order to (i) diagnose acute kidney injuries, (ii) prevent further degradation of renal function and (iii) adjust the dose of several renally excreted drugs. GFR estimation is usually based on serum creatinine (SCr) which is known to be a rather insensitive GFR biomarker. First, SCr is unable to reflect rapidly changing GFR, i.e. in a non steady state [1]. Moreover, SCr is not exclusively cleared by glomerular filtration but is also partially secreted by renal tubules. This well-known phenomenon may account for substantial GFR overestimation. Even more concerning in the context of ICU is the dependency of SCr to muscular mass [2]. Critically ill patients are particularly prone to alteration in their muscle mass not only occurring during their stay in the ICU but also frequently already present at their admission to the ICU.

As compared to SCr, serum cystatin C (SCysC) is less dependent to muscle mass and is deemed as a more accurate GFR biomarker in different situations [3,4]. Several studies performed in critically ill patients have suggested the superiority of SCysC for the detection of a decreased GFR [5-9]. None of those studies has however compared the performance of SCysC against a reference method of GFR measurement. The real advantage of SCysC over SCr to estimate the so-called “true” GFR still remains to be demonstrated in the specific context of ICU.

In this study, we directly assessed the true GFR of patients hospitalized in ICU by measuring their urinary clearance of iohexol [10]. The corresponding measured value was used as a gold standard to compare the relative performance of SCr and SCysC as GFR biomarkers in critically ill patients to detect decreased GFR. In this specific population, several studies have suggested that equations based on SCr are both inaccurate and imprecise [11-13]. We also study the ability of SCr-, ScysC-based and combined equations to estimate GFR in these ICU patients [14].

## Methods

### Subjects

Patients from 3 University hospitals (CHU Sart Tilman, University of Liège, Liège, Belgium; Hôpital Nord, University Jean Monnet, Saint Etienne, France and University of Clermont-Ferrand, Clermont-Ferrand, France), were included if they were older than 18 years, hemo-dynamically stable (mean arterial blood pressure ≥ 65 mmHg with no modification in vaso-active drugs within the last 12 hours), under mechanical ventilation, with a urinary catheter, with a minimal diuresis of 400 mL in the last 6 hours, and with SCr < 133 μmol/L (1.5 mg/dL). This value is arbitrary and recomended by the international guidelines of the K-DIGO (for “Kidney Disease: Improving Global Outcomes”). The main objective was to study the performance of SCr (and new biomarkers) to detect GFR below 60 mL/min. The sensitivity of SCr is clearly insufficient but its specificity is good. Therefore, we designed a study to try and address clinically relevant questions. In clinical practice and in the context of the detection of decreased GFR, there is few doubt that a given patient with SCr above 1.5 mg/dL suffered from kidney disease. Patients were not included in case of liver dysfunction (prothrombin time < 50%), pregnancy, history of allergic reaction to iodine, active dysthyroidism and necessity of anti-inflammatory treatment by steroids. This study was approved by the Ethics Committee of CHU Sart Tilman (Liège, Belgium) and Hôpital Nord (Saint-Etienne, France). The Belgian number of this study is BE7072006347. The French number is 2009-010710-29. The necessary written informed consent was obtained for all patients involved in the study, including consent to publish.

### GFR measurement and biomarkers

GFR was determined by urinary clearance of iohexol (Omnipaque 240 mgI/mL, Amersham Health, New Jersey, USA). Two hours after the injection of 10 milliliters of iohexol, 4 clearances periods of one hour each were performed with plasma samples drawn at the beginning and the end of each period. Urine was separately collected for each period. Clearances periods for which the result was outside ± 20% of the mean clearance, or for which urine outflow was lower than 20 milliliters were not retained for the analysis. Iohexol clearance was determined as the mean of the different clearances periods. Iohexol samples were assayed by high performance liquid chromatography with high analytical performances [15]. External quality control was provided by Equalis (Uppsala, Sweden).

SCr was measured by the IDMS traceable enzymatic method (Roche Diagnostics, Mannheim, Germany) on Modular apparatus. SCysC was measured by a particle-enhanced nephelometric immunoassay on the BNII nephelometer (Siemens Healthcare Diagnostics, Marburg, Germany). The assay was calibrated against the international certified reference material ERM-DA471/IFCC for cystatin C. Both SCr and SCysC were sampled at the beginning of the first clearance period, kept at -80°C to be centrally processed at the University of Liège (Department of Clinical Chemistry, ISO 15189 Standard-accredited laboratory).

We studied the performance of the most widely used SCr- and ScysC-based equations, i.e. the Modification of Diet in Renal Disease (MDRD) [16] and the CKD-Epidemiology Collaboration (CKD-EPI) equations (Table 1) [14].

**Table 1:** GFR estimates (SCr serum creatinine, SCysC serum cystatine C)

**Basis of equation and sex**	**SCr and SCysC values**	**Equation for estimating GFR**
MDRD		175 × Scr^-1.154^ × age^-0.203^ × [0.742 if female]
CKD-EPI SCr		
Female	SCr ≤ 0.7 mg/dL	144 × (Scr/0.7)^-0.329^ × 0.993^age^
	SCr > 0.7 mg/dL	144 × (Scr/0.7)^-1.209^ × 0.993^age^
Male	SCr ≤ 0.9 mg/dL	141 × (Scr/0.9)^-0.411^ × 0.993^age^
	SCr > 0.9 mg/dL	141 × (Scr/0.9)^-1.209^ × 0.993^age^
CKD-EPI SCysC	SCysC ≤ 0.8 mg/L	133 × (SCysC/0,8)^-0.499^ × 0.996^age^ [×0.932 if female]
	SCysC >0.8 mg/L	133 × (Scys/0,8)^-1.328^ × 0.996^age^ [×0.932 if female]
CKD-EPI combined		
Female	SCr ≤ 0.7 mg/dL and SCysC ≤ 0.8 mg/L	130 × (Scr/0.7)^-0.248^ × (SCysC/0.8)^-0.375^ × 0.995^age^
	SCr ≤ 0.7 mg/dL and SCysC > 0.8 mg/L	130 × (Scr/0.7)^-0.248^ × (SCysC/0.8)^-0.711^ × 0.995^age^
	SCr > 0.7 mg/dL and SCysC ≤ 0.8 mg/L	130 × (SCr/0.7)^-0.601^ × (SCysC/0.8)^-0.375^ × 0.995^age^
	SCr >0.7 mg/dL and SCysC ≥ 0.8 mg/L	130 × (SCr/0.7)^-0.601^ × (SCysC/0.8)^-0.711^ × 0.995^age^
Male	SCr ≤0.9 mg/dL and SCysC ≤ 0.8 mg/L	135 × (SCr/0.9)^-0.207^ × (SCysC/0.8)^-0.375^ × 0.995^age^
	SCr ≤ 0.9 mg/dL and SCysC > 0.8 mg/L	135 × (SCr/0.9)^-0.207^ × (SCysC/0.8)^-0.711^ × 0.995^age^
	SCr >0.9 mg/dL and SCysC ≤ 0.8 mg/L	135 × (SCr/0.9)^-0.601^ × (SCysC/0.8)^-0.375^ × 0.995^age^
	SCr >0.9 mg/dL and SCysC > 0.8 mg/L	135 × (SCr/0.7)^-0.601^ × (SCysC/0.8)^-0.711^ × 0.995^age^

### Statistics

Data were expressed as mean ± standard deviation (SD) when distribution was normal and as median and interquartile range [IQR] if not. Performance of SCr and SCysC was evaluated by analyzing their respective correlation with iohexol clearance and by calculating the area under the ROC curves to detect an iohexol clearance below 60 mL/min. Statistics were performed using MedCalc® (MedCalc Software, Mariakerke, Belgium). The predictive performance of the GFR estimates was assessed with the following parameters:

– Absolute bias, defined as the mean difference between estimating GFR (eGFR) and measured GFR (mGFR), a negative value meaning that eGFR under-estimates true GFR.

– Precision, evaluated by the standard deviation of the mean difference between eGFR and mGFR

– Accuracy, defined as the proportion of eGFR values within +/- 30% of the mGFR.

Comparison of precision and accuracy were performed using F-test and McNemar paired test.

A p value < 0.05 was considered as statistically significant.

## Results

**Figure 1:**
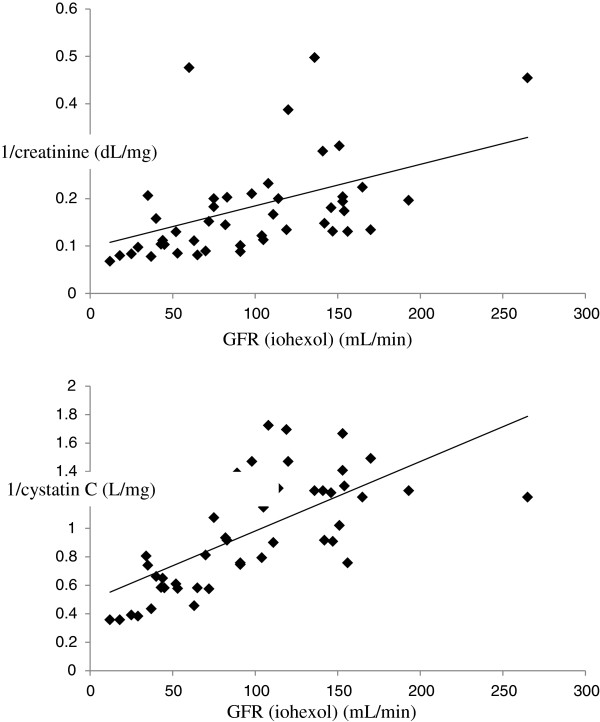
**Correlations between the inverse of creatinine and GFR (upper) (y = 0,09024 + 0,0009156×) and the inverse of cystatin C and GFR (lower) (y = 0,4939 + 0,004871×).**

**Figure 2:**
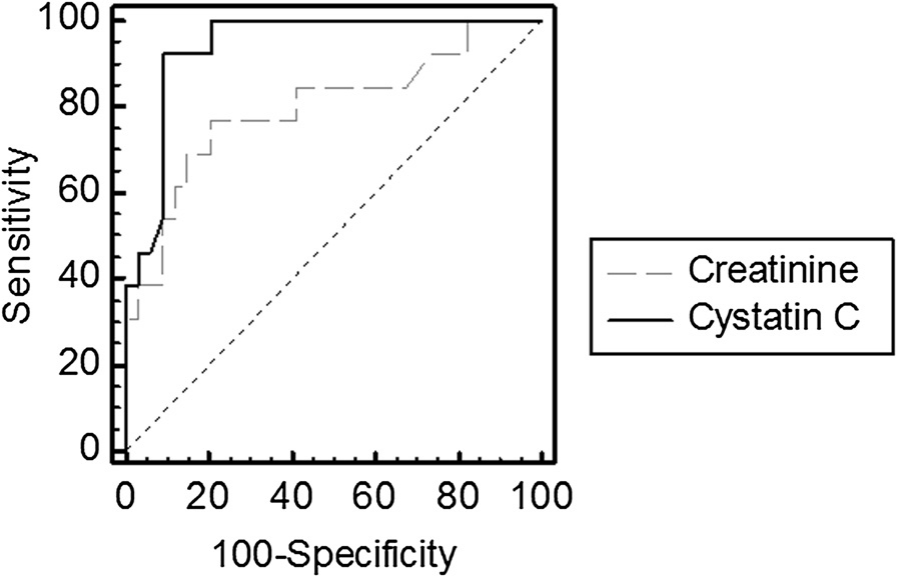
**ROC curves analysis for cystatin C (―) (AUC** = **0.942) and creatinine (----) (AUC** = **0.799) to detect GFR under 60 mL/min (p** = **0.014).**

Fifty-one patients were included. All patients were Caucasian. Four patients were retrospectively excluded due to technical errors in the clearance procedure. According to our criteria, 86% of clearance periods have been considered. Mean GFR value in our population was 98 ± 56 mL/min. Characteristics of the 47 patients retained for the analysis are presented in Table 2. While mean SCr was low (0.7 ± 0.33 mg/dL), 28% of patients had an iohexol clearance below 60 mL/min/1.73 m^2^. The reciprocal of SCysC better correlated to iohexol clearance than the reciprocal of SCr (r = 0.667 and r = 0.499, respectively) (Figure 1). This difference did not reach the statistical significance. SCysC was however significantly superior to SCr to discriminate patients with an iohexol clearance above or below the threshold of 60 mL/min (AUC ROC of 0.942 and 0.799, respectively, p = 0.014) (Figure 2).

**Table 2:** Clinical and biological characteristics of the population

	
Age (years)	62 ± 17
Gender	25 women/ 22 men
Weight (kg)	81 ± 24
Height (cm)	168 ± 10
Body mass index (kg/m^2^)	29 ± 8
Cause of admission	Sepsis (39%)
	Neurologic diseases (32%)
	Trauma (16%)
	Myocardial infarction (5%)
	Other (8%)
SOFA (Sepsis-related Organ Failure Assessment) score	5 [4;9]
GFR (mL/min)	96 ± 54
Serum creatinine (mg/dL)	0.70 ± 0.33
Serum cystatin C (mg/L)	1.26 ± 0.61

The global performances of estimating GFR were very poor (Table 3). The best accuracy within 30% was observed both for the CKD EPI SCr and combined equations (59 and 62%, respectivelly). Performance of the MDRD was significantly worse (40%).

**Table 3:** Predictive performances of the MDRD, CKD-EPI SCr, CKD-EPI SCysC, and combined equations in ICU patients

**GFR estimates**	**Bias (mL/min)**	**Absolute Precision mL/min**	**Accuracy 30%**
MDRD	+35	70	40
CKD-EPI	+ 1	37	60*
CKD-EPI Scyst	-26	36	53
CKD-EPI combined	-12	35	62

*: p < *0.05 versus MDRD study equation.*

## Discussion

SCysC is recognized to be far less influenced by muscular mass than SCr [4]. Not surprinsingly, SCysC has been validated as a superior GFR biomarker to detect chronic kidney disease (defined as GFR below 60 mL/min/1.73 m^2^) in several specific populations with decreased or abnormal muscular mass like in the elderly, cirrhotic patients, renal transplant patients, and patients suffering from anorexia nervosa [3,13,17]. Herein, we extend this notion to critically ill patients. Numerous previous studies performed in severely ill patients (not necessarily hospitalized in ICU) have suggested the better performance of SCysC for detecting AKI [5,6]. However since most of those studies did not provide a reference GFR measurement against which SCr and SCysC could be thoroughly compared, the real added value of SCysC as a marker of GFR has remained partly speculative [6]. Among all these studies, only two of them realized after cardiac surgery have measured GFR by a reference method (iohexol or ^51^Cr-EDTA) and showed superior performance of cystatin C [18,19]. Focusing specifically on critically ill patients, Le Bricon *et al.* measured GFR by a reference method (^51^Cr-EDTA) and showed a better correlation between 1/cystatin C and GFR than between 1/creatinine and GFR (r = 0.755 versus r = 0.686). The authors also demonstrated a better sensitivity-specificity of cystatin C to detect impaired GFR [20]. However, the number of patients included in this study was very limited (n = 15). In addition, in this study, the ^51^Cr-EDTA clearance was carried out according to a plasmatic method which is not necessarily as accurate as the urinary method especially in patients with variable and unpredictable volumes of distribution [21].

As compared to the aforementioned studies, the major strength of ours is the use of a very rigorous method of GFR measurement in a selected population of stable but critically ill patients. In this regard, our results do really reflect the physiological performances of the two biomarkers against the “true” GFR, a parameter that is particularly uneasy to approach in the context of ICU. Thus, our data strongly suggest the superiority of SCysC over SCr in critically ill patients.

However, even if ScysC is better than SCr to detect decreased GFR, the accuracy, and especially the precision, of ScysC-based or combined equations to estimate GFR is clearly insufficient and have no added value compared to SCr-based equations. This result is explained by the different characteristics of the subject compared to the populations analyzed in the CKD-EPI cohort (chronic versus acute disease, decreased muscular mass etc).

There are however several limitations to our study. First, we enrolled prevalent patients and our study is purely cross-sectional. Although patients with relatively low SCr have been included in our study, we cannot exclude the hypothesis that patient with pre-existing chronic kidney disease, have been included. Second, our sample remains relatively limited and only includes patients with SCr < 133 μmol/L (1.5 mg/dL). Our results must be confirmed in a larger cohort and in patients with a larger range of SCr and GFR. Lastly, cystatin C concentration is also influenced by non-GFR factors. While we took into account some confounding factors (steroids and dysthyroidism), others factors may have played a role [22,23]. Finally, the better performance of ScysC could potentially be due to a quicker reach of its steady state when GFR rapidly changes. However, this point remains purely speculative and cannot be addressed in our cross- sectional designed study.

## Conclusion

In conclusion, SCysC performs significantly better than SCr in order to detect critically ill patients with measured GFR below 60 ml/min. More generally, we believe that SCysC is a more valid GFR biomarker than SCr in ICU and as such, might be evaluated as part of the AKI definition/classification in replacement of SCr.

### Key messages

– High percentage of patients hospitalized in intensive care units has normal creatinine concentration but decreased glomerular filtration rate.

– Cystatin C is better than creatinine to detect measured glomerular filtration rate less than 60 mL/min/1.73 m^2^.

– All estimating equations lack of precision to estimate GFR.

## Abbreviations

GFR: Glomerular filtration rate; ICU: Intensive care unit; SCr: Serum creatinine; SCysC: Serum cystatin C; SOFA: (Sepsis-related Organ Failure Assessment).

## Competing interest

The authors declare that they have no competing interest.

## Authors’ contribution

PD, EC, CM are the principal investigators. PD and CM have been involved in drafting the manuscript. EC is the Biochemist who measured serum creatinine and cystatin C. JM, MM, NM, GC, BL, BED, PDa, AL and CM are the clinicians who included the patients in the different centers. They all participated to the acquisition of the data. BL, PDa, JMK, AL and CM have critically corrected the manuscript as Chief of the Department of medical intensive care (Liège), general intensive care (Liège), Nephrology (Liège), general intensive care (Clermont Ferrant) and nephrological intensive care (Saint-Etienne), respectively. All authors read and approved the final manuscript.

## Pre-publication history

The pre-publication history for this paper can be accessed here:

http://www.biomedcentral.com/1471-2369/15/9/prepub
